# Effect of basil seed and xanthan gum on physicochemical, textural, and sensory characteristics of low‐fat cream cheese

**DOI:** 10.1002/fsn3.3542

**Published:** 2023-07-04

**Authors:** Jalal Portaghi, Ali Heshmati, Mehdi Taheri, Ebrahim Ahmadi, Amin Mousavi Khaneghah

**Affiliations:** ^1^ Department of Nutrition and Food Hygiene, School of Medicine Nutrition Health Research Center, Hamadan University of Medical Sciences Hamadan Iran; ^2^ Department of Biosystems Engineering Bu‐Ali Sina University Hamedan Iran; ^3^ Department of Fruit and Vegetable Product Technology Prof. Wacław Dąbrowski Institute of Agricultural and Food Biotechnology – State Research Institute Warsaw Poland; ^4^ Department of Technology of Chemistry Azerbaijan State Oil and Industry University Baku Azerbaijan

**Keywords:** basil seed gum, cream cheese, response surface method, texture analysis, xanthan

## Abstract

This study aims to produce fat‐reduced cream cheese using the different levels (0.25%–0.5%) of basil seed and xanthan gum by a RSM method. The basil seed, xanthan gum, and fat levels did not significantly influence the cream cheese's pH and acidity. With the fat reduction, textural properties were lost; for example, hardness, gumminess, and adhesiveness increased, and cohesiveness decreased. In addition, low‐fat cream cheese's sensory score (taste, mouthfeel, and overall acceptance score) was lower. However, adding basil seed and xanthan gum could improve water holding capacity (WHC), hardness, gumminess, cohesiveness, adhesiveness and scores of mouthfeel, and overall acceptance. Basil seed gum had a better impact than xanthan on fat‐reduced cream cheese properties among the two gums. In general, results showed that adding 0.5% basil and 0.5% xanthan into cream cheese could manufacture a product with a reduced‐fat level (19.04%). At the same time, its physicochemical, sensory, and textural attributes were similar to cream cheese with high fat (24%). In addition, the price of the obtained product was lower.

## Graphical abstract

Basil seed and xanthan gum were used to produce fat‐reduced cream cheese by the RSM method. Basil seed and xanthan gum could improve attributes of fat‐reduced samples. Fat‐reduced samples. (19.04%) have similar properties to high‐fat specimens (24%). The price of the obtained product was lower.

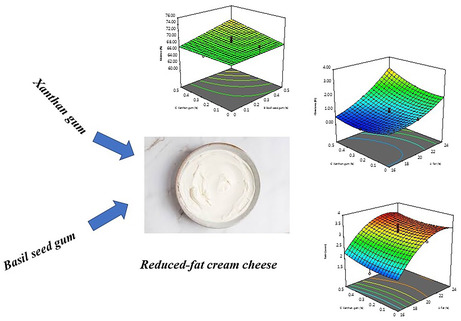

## 1 INTRODUCTION

People's extended awareness and knowledge about fitness and healthy lifestyle have increased demand for low‐calorie foodstuffs, including reduced‐fat products (Amiri et al., 2021; Chailangka et al., 2023; Isaacs, 2019; Khanal & Bansal, 2020; Naghshi et al., 2022; Syan et al., 2022). Excessive fat intake has different harmfulness and causes various illnesses, including obesity, cardiovascular disease, and different types of cancers (Koene et al., 2016; Schwab et al., 2014).

Dairy products are a good source of protein, minerals, vitamins, and fatty acids for humans (Mirmahdi et al., 2021; Nejad et al., 2019; Yu et al., 2022). However, due to the impact of full‐fat dairy products on the increment of blood cholesterol and saturated fatty acids (SFAs), current US and Canadian dietary guidelines for cardiovascular health suggest the consumption of low‐fat dairy products (Hirahatake et al., 2020; Mozaffarian, 2019; Naghshi et al., 2022). The epidemiologic studies indicated the association of low‐fat dairy product intake with lower risk of metabolic syndrome incidences such as type 2 diabetes, blood pressure, and the improvement of lipid profile (Drouin‐Chartier et al., 2016; Khodadadi et al., 2017; Margolis et al., 2011).

Cream cheese is one of the most widely used dairy products and has a soft and smooth texture with a mildly acidic flavor (Brighenti et al., 2018; Pombo, 2021; Popescu et al., 2023). It is a type of unripened soft cheese produced from a mixture of cream and milk or skim milk and contains a high‐fat level (Ong et al., 2020; Surber et al., 2021). There is a high trend for low‐fat cream cheese production and consumption today.

The lower moisture level in non‐fat solids, free fat, and proteolysis activity, and the great content of protein in low‐fat cream cheese could cause defects such as weak flavor, structure and texture, and low acceptability (Aydinol & Ozcan, 2018). To improve low‐fat cheeses' textural and sensory properties, some methods, such as homogenization, the addition of capsule‐forming bacteria cultures, and modifications to process conditions were applied (Khanal & Bansal, 2020). In recent years, the utilization and incorporation of fat alternatives, macromolecules with similar physical and chemical properties to triglycerides, and protein and carbohydrate‐based fat replacers into cheese were recommended to resolve some mentioned defects (Hammam & Ahmed, 2019). Fat replacers showed different functional attributes that may improve the structure and sensory characteristics of low‐fat cheeses (Syan et al., 2022).

Basil seed gum is a surface‐active hydrocolloid with stabilizing and emulsifying properties obtained from *Ocimum basilicum* L. seeds (Razavi & Naji‐Tabasi, 2023; Yang et al., 2021). In the current years, basil seed gum utilization has increased (Ghasempour et al., 2020; Hosseini‐Parvar et al., 2015; Lee & Chin, 2017; Naji‐Tabasi & Razavi, 2017; Saengphol & Pirak, 2018; Sara Naji‐Tabasi, 2016). The application of basil seed gum along with other gums such as xanthan, Κ‐carrageenan and guar as fat‐replacer in some products was considered (Biglarian et al., 2021, 2022; Hesarinejad et al., 2021; Shamsaei et al., 2017). However, no published reports in the literature about the simultaneous utilization of basil seed gum and xanthan as fat‐replacer in low‐fat cream cheese are available. Adding both the mentioned gums into cream cheese could influence product properties. This study aimed to optimize the physicochemical, textural, and sensory properties of low‐fat cream cheese containing basil seed and xanthan gum.

## 2 MATERIALS AND METHODS

### 2.1 Materials

Milk (3% fat) and animal cream (40%) were obtained from Malayer Cheese, cream company (Malayer, Hamadan, Iran), and starter cultures containing *Streptococcus thermophilus* and *Lactococcus lactis* subsp. *cre*atures were bought from Chr.Hansen (Copenhagen, Denmark). Basil seed gum (powdered form) was purchased from Reyhan Gum Parsian Inc. (Tehran, Iran), and xanthan gum was bought from Danisco (Copenhagen, Denmark). Other chemicals were from Merck (Darmstadt, Germany).

### 2.2 Cream cheese preparation

Two types of cream cheese were produced: control cream cheese samples (without basil seed and xanthan gum) and reduced low‐fat cream cheese samples containing various levels (0–0.5%) of basil seed and/or (0–0.5%) xanthan gum. For preparing control cream cheese, milk was pasteurized (74–78°C for 16 s) and ultrafiltrated. The obtained retentate or condensed milk is mixed with the required amount of animal cream containing 40% fat, pasteurized (76–78°C for 60 s), and homogenized (at a pressure of 50–70 bar). Then, the temperature of the retentate reached 30°C, and starter culture and rennin were added. When the pH of the samples reached 4.8, sodium chloride (0.7%) was added to deactivate the mesophilic and thermophilic bacteria and stop the fermentation process. After ensuring the suitable mixing of the salt with the curd, samples were homogenized (at a pressure of 150–200 bar) and pasteurized (76°C and 1 min), and packaged and stored (4–6°C).

For the production of low‐fat cream cheese samples, basil seed and xanthan gum were added to the curd at the same time. Other steps are similar to the preparation of the control cream cheese sample.

### 2.3 Physicochemical properties

For the assessment of cheese's physicochemical properties, AOAC official method was utilized (Horwitz, 2010). The acidity was determined as titratable acidity (AOAC 947.05). Fat content was determined by the Rosse–Gottlieb method (AOAC 933.05). Protein level was obtained by measuring total nitrogen using the Kjeldahl method (AOAC 920.123). Moisture was measured by gravimetric method (AOAC 935.42). A digital pH meter (Metrohm, Herisau, Switzerland) determined pH.

To measure the water holding capacity (WHC), 5 g of cream cheese samples is poured into a falcon tube and centrifuged (4058 × g for 20 min at 8°C). The supernatant solution was decanted, and the precipitate was weighed. Finally, WHC is calculated according to the following Equation 1 (Mousavi et al., 2019a, 2019b):

(1) $$\begin{array}{c} \mathrm{WHC}\\ =\left(\text{the weight of precipitate}/\text{the initial weight of cream cheese}\right)\times 100 \end{array}$$

### 2.4 Textural profile analysis

Textural parameters, including hardness, gumminess, cohesiveness, and adhesiveness, were determined by using texture profile analysis (TPA) (Zwick Company, Ulm, Germany) according to the previous study (Mousavi et al., 2019a, 2019b). For texture analysis, the back‐extrusion test in 4 cycles was applied. The cylindrically shaped probe with a diameter of 40 mm penetrated samples until the depth of 25 mm, with a rate of 10 mm/s.

### 2.5 Sensory evaluation

After 1 day of cold storage of cream cheese samples, a five‐point hedonic scale test was utilized for sensory evaluation (Mousavi et al., 2019a, 2019b). This experiment was approved by the Ethical Committee of the Hamadan University of Medical Science (Ethical code: IR.UMSHA.REC.1399.527). Fifty people were non‐trained panel test samples. One hundred grams of each sample was presented to each person to assess it for taste, mouthfeel, appearance, and overall acceptability.

### 2.6 Experimental designs and analysis of data

The response surface method (RSM) was utilized to show the influence of various independent attributes of produced cream cheese samples. Independent variables were fat levels (X_1_), basil seed gum concentration (X_2_), and xanthan gum concentrations (X_3_); responses were pH, acidity, protein, moisture, WHC, hardness, gumminess, cohesiveness, adhesiveness, taste, mouthfeel, appearance, and overall acceptability obtained from design expert software contained 20 runs. The variables chosen for basil seed gum and xanthan gum concentration were 0%–0.5%, while for fat content was 16%–24%. The response of dependent variables in each independent variable level is shown in Table 1.

**TABLE 1:** Experimental design and results of dependent variables.

Run	Independent variables			Dependent variables												
	Fat content (%)	Basil seed gum content (%)	Xanthan gum content (%)	pH	Acidity (%)	Protein (%)	Moisture (%)	WHC (%)	Hardness (*N*)	Gumminess (*N*)	Cohesiveness (*N*)	Adhesiveness (*N*)	Taste (score)	Mouthfeel (score)	Appearance (score)	Overall acceptability (score)
1	20	0.5	0.25	4.71	0.75	5.5	68.87	98.34	1.62	1.60	0.63	19.56	2.60	3.40	5.00	3.60
2	16	0.25	0.25	4.76	0.75	5.79	73.65	88.69	3.66	2.80	0.44	25.23	1.80	2.00	4.80	2.90
3	16	0.5	0.5	4.75	0.68	5.76	74.25	95.15	2.03	1.91	0.67	20.2	2.40	2.80	5.00	3.30
4	20	0.25	0.5	4.68	0.73	5.5	68.25	100	3.64	2.75	0.70	25.23	3.20	3.20	4.80	3.80
5	20	0.25	0.25	4.7	0.80	5.36	69.23	94.35	3.19	2.69	0.52	24.23	3.40	3.00	4.80	3.70
6	16	0	0.5	4.76	0.69	5.97	72.56	90.23	5.8	4.33	0.49	40.55	2.00	1.20	4.80	2.71
7	24	0.25	0.25	4.79	0.66	4.87	63.05	98.4	2.2	2.12	0.64	20.98	3.20	3.20	5.00	3.72
8	20	0.25	0.25	4.71	0.77	5.32	69.16	96.89	2.92	2.50	0.59	24.17	3.10	3.30	4.80	3.70
9	20	0.25	0	4.67	0.72	5.24	70.16	91.41	2.75	2.50	0.76	23.95	3.20	2.60	4.80	3.60
10	24	0.5	0	4.77	0.57	4.79	62.56	100	1.41	1.10	0.69	19.23	3.40	3.60	5.00	4.00
11	20	0.25	0.25	4.7	0.85	5.17	69.41	93.23	2.7	2.40	0.48	22.65	3.50	3.10	4.80	3.90
12	20	0.25	0.25	4.69	0.73	5.1	70.26	94.23	2.63	2.35	0.55	22.63	3.20	3.40	4.90	3.80
13	20	0.25	0.25	4.72	0.67	5.08	69.98	97.56	2.55	2.29	0.62	21.73	3.60	2.90	5.00	3.80
14	20	0	0.25	4.67	0.75	5.05	67.31	95.23	5.38	3.55	0.71	37.65	3.20	2.00	4.80	3.40
15	24	0	0.5	4.75	0.57	4.23	62.15	99.1	4.77	3.40	0.76	35.73	3.20	1.60	4.80	3.20
16	20	0.25	0.25	4.69	0.73	5	68.15	96.8	2.48	2.23	0.56	21.32	3.30	2.90	5.00	3.70
17	24	0.5	0.5	4.73	0.72	4.5	69.31	100	1.21	0.50	0.89	14.23	3.60	2.80	5.00	3.70
18	16	0	0	4.74	0.80	5.7	71.98	62.4	7.35	3.75	0.36	38.74	2.00	1.00	4.80	2.60
19	24	0	0	4.74	0.75	4.05	61.45	91.23	4.13	2.90	0.58	26.05	3.20	4.40	4.80	4.10
20	16	0.5	0	4.72	0.59	5.63	72.32	91	1.9	1.79	0.51	19.65	1.80	1.60	5.00	2.70

The analysis of variance (ANOVA) and the determination of model, lack‐of‐fit, pure error, and other statistical calculations shown in Table 2 were performed by Software of Design‐Expert version 12.0.0 (Version 12; Stat‐Ease Inc., Minneapolis, MN, USA)

**TABLE 2:** ANOVA for responses by response surface method.

Response	Source	Sum of square	df	Mean square	*F* value	Probe > *F*	Model
pH	Model	0.0203	9	0.0023	10.12	0.0006	Quadratic
	Lack of fit	0.0016	5	0.0003	2.27	0.1948	
	Pure error	0.0007	5	0.0001			
	*R* ^2^						0.9011
Acidity (%)	Model	0.0812	9	0.009	3.65	0.0279	Quadratic
	Lack of fit	0.0050	5	0.0010	0.2544	0.9204	
	Pure error	0.0197	5	0.0039			
	*R* ^2^						0.7668
Protein (%)	Model	4.28	3	1.43	30.69	<0.0001	Linear
	Lack of fit	0.6429	11	0.0584	2.91	0.1242	
	Pure error	0.1005	5	0.0201			
	R^2^						0.8520
Moisture (%)	Model	249.12	6	41.52	26.89	<0.0001	Linear
	Lack of fit	17.35	8	2.17	3.99	0.0720	
	Pure error		2.72	5	0.5436		
	*R* ^2^						0.9254
WHC (%)	Model	1185.18	8	131.69	16.02	<0.0001	Quadratic
	Lack of fit	66.23	5	13.25	4.15	0.2335	
	Pure error	15.95	5	3.19			
	*R* ^2^						0.9352
Hardness (*N*)	Model	46.16	9	5.13	28.42	<0.00001	Quadratic
	Lack of fit	1.45	5	0.2906	4.13	0.0730	
	Pure error		0.3522	5	0.0704		
	*R* ^2^						0.9624
Gumminess (*N*)	Model	14.71	6	2.45	88.18	<0.0001	Linear
	Lack of fit	0.2243	8	0.0279	1.01	0.5119	
	Pure error	0.1381	5	0.0276			
	*R* ^2^						0.9760
Cohesiveness (*N*)	Model	0.1787	3	0.0596	7.82	0.0020	Linear
	Lack of fit	0.1096	11	0.01	4.03	0.0678	
	Pure error	0.0124	5	0.0025			
	*R* ^2^						0.5944
Adhesiveness (*N*)	Model	952.37	9	105.82	27.59	<0.0001	Quadratic
	Lack of fit	31.05	5	6.21	4.25	0.0692	
	Pure error	7.31	5	1.46			
	*R* ^2^						0.9613
Taste (Score)	Model	6.26	9	0.6955	9.27	0.0009	Quadratic
	Lack of fit	0.5753	5	0.1151	3.29	0.1087	
	Pure error	0.175	5	0.035			
	*R* ^2^						0.8930
Mouthfeel (Score)	Model	13.25	9	1.47	12.47	0.0002	Quadratic
	Lack of fit	0.9654	5	0.1931	4.39	0.0652	
	Pure error	0.22	5	0.044			
	*R* ^2^						0.9179
Appearance (Score)	Model	0.1040	3	0.0347	6.81	0.0036	Linear
	Lack of fit	0.0332	11	0.0030	0.3119	0.9501	
	Pure error	0.0483	5	0.0097			
	*R* ^2^						0.5606
Overall acceptability (Score)	Model	3.51	9	0.3901	30.97	<0.0001	Quadratic
	Lack of fit	0.0983	5	0.0197	3.55	0.0954	
	Pure error	0.0277	5	0.0055			
	*R* ^2^						0.9654

### 2.7 Optimization

For optimization, responses such as protein content, moisture content and cohesiveness, and WHC and taste, mouthfeel, appearance, and overall acceptability scores were selected at maximum value and hardness, adhesiveness, and gumminess at minimum level. Other dependent variables, such as pH, acidity, and independent variables, basil seed gum and xanthan gum concentration, and fat amount, were placed within the range (between lower and higher levels).

## 3 RESULTS AND DISCUSSION

### 3.1 Physico‐chemical properties

There is no significant difference in pH and acidity of various samples. Other authors reported similar results (Akin & Kirmaci, 2015; Esen & Güzeler, 2023; Kavas et al., 2004).

The protein content of cream cheese samples ranged from 4.05% to 5.97%. The highest amount of protein (5.97%) was observed in samples without basil and xanthan gum containing 16% fat. While the lowest amount of protein (4.05%) was observed in samples without basil seed and xanthan gum containing 24% fat. The findings showed that basil and xanthan gum had no significant effect on protein, although the amount of fat had a significant effect (*p* < .05) on protein. Collectively, protein content followed the linear model, and the lack of fit of this model was insignificant.

**FIGURE 1:**
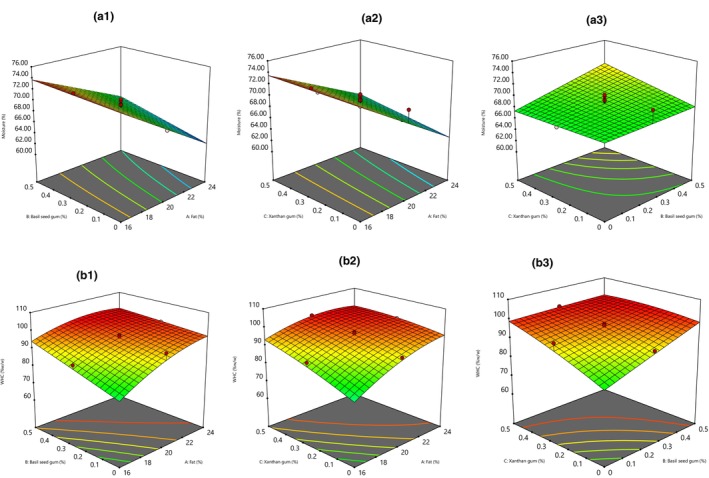
Response surface plot of the effects of basil seed and xanthan gum and fat content on moisture content (a1, a2, and a3) and water holding capacity (WHC, b1, b2, and b3) of cream cheese.

The moisture content of cream cheese containing a high basil seed and xanthan gum was greater, while moisture content had an opposite relationship with fat value. The highest moisture value (74.25%) was related to cheese samples containing 16% fat, 0.5% basil seed, and 0.5% xanthan gum. The results showed that the fat and basil seed gum content had a significant effect (*p* < .05) on moisture, although the xanthan gum effect was insignificant (Figure 1a1–a3). Experimental results demonstrated that moisture content followed the quadratic polynomial model (Table 3):

(2) $$\begin{array}{c} \text{Moisture content}=68.70–4.62X_{1}+1.19X_{2}+0.80\text{5X3}+0.78{\text{1X}}_{1}X_{2}\\ +0.6175\;X_{1}X_{3}+0.9250X_{2}X_{3} \end{array}$$

**TABLE 3:** Regression coefficients of coded factors for the responses.

Response	Intercept (X_0_)	Variable																	
		X_1_	*p* value	X_2_	*p* value	X_3_	*p* value	X_1_X_2_	*p* value	X_1_X_3_	*p* value	X_2_X_3_	*p* value	$X_{1}^{2}$	*p* value	$X_{2}^{2}$	*p* value	$X_{3}^{2}$	*p* value
pH	4.7	0.005	.3115	0.002	.6811	0.003	.5398	0.005	.3663	−0.01	.0877	−0.005	.6363	0.0764	<.0001	−0.0086	.3655	−0.0236	.0255
Acidity (%)	0.734	−0.024	.15766	−0.025	.14269	−0.004	.80422	0.0238	.20619	−0.0013	.94468	0.0663	.00366	−0.0505	.12312	−0.0055	.20619	−0.0305	.3334
Protein (%)	5.18	−0.6410	<.0001	0.118	.1027	0.055	.4316	–	–	–	–	–	–	–	–	–	–	–	–
Moisture (%)	68.70	−4.624	<.0001	1.186	.0099	0.805	.0612	0.78	.0992	0.6175	.1833	0.925	.0552	–	–	–	–	–	–
WHC (%)	96.07	6.13	<.0001	4.63	.0005	4.84	.0003	−2.98	.0148	−3.01	.0140	−3.94	.0030	−3.37	.0795	−0.1345	.9355	−1.21	.4984
Hardness (*N*)	2.83	−0.702	.0004	−1.926	<.0001	−0.009	.9479	0.3675	.0345	0.2325	.1527	0.105	.5005	−0.0186	.9435	0.5514	.0345	0.2464	.359
Gumminess (*N*)	2.47	−0.4558	<.0001	−1.103	<.0001	0.0859	.1272	−0.04	.5091	−0.1012	.1099	−0.1947	.0057	–	–	–	–	–	–
Cohesiveness (*N*)	0.6074	0.1089	.001	0.0486	.0975	0.0604	.0439	–	–	–	–	–	–	–	–	–	–	–	–
Adhesiveness (*N*)	23.36	−2.815	.0011	−8.585	<.0001	0.832	.2088	1.39	.0725	0.29	.6842	−1.9925	.0165	−1.1109	.3691	4.3891	.0725	0.3741	.7579
Taste (Score)	3.23	0.66	<.0001	0.02	.822	0.08	.3774	0.05	.6169	−0.05	.6169	0.1	.3261	−0.5591	.0069	−0.1591	.6169	0.1409	.4136
Mouthfeel (Score)	3.05	0.7	<.0001	0.4	.0043	0.16	.1724	−0.225	.0943	−0.625	.0004	0.375	.0116	−0.3636	.1104	−0.2636	.2329	−0.0636	.7655
Appearance (Score)	4.88	0.02	.0036	0.1	.3887	0.0001	1	–	–	–	–	–	–	–	–	–	–	–	–
Overall acceptability (Score)	3.72	0.434	<.001	0.12	.007	−0.04	.286	−0.04	.3372	0.24	.0001	0.1425	.0049	3250	.0007	0.155	.045	0.045	.5211

*Note*: X_1_, Fat content (%); X_2_, Basil seed gum content (%); X_3_, Xanthan gum content (%).

Our finding was similar to Jooyandeh et al. (2017). They observed that fat reduction in Iranian White cheese containing Persian and almond gums was accompanied by a significant increase in moisture and protein amounts (Jooyandeh et al., 2017).

There were significant differences in WHC among various samples (*p* < .05). The findings of design expert software showed that a quadratic model could significantly predict the WHC as a function of fat, basil seed, and xanthan gum amount. This model is well fitted (*p* < .001, *F*‐value = 16.02) and also showed a nonsignificant lack of fit (Table 2). The equation for WHC included the following:

(3) $$\begin{array}{c} \mathrm{WHC}\;\left(\%\right)=96.07+6.13X_{1}+4.63X_{2}+4.84X_{3}-2.98X_{1}X_{2}\\ -3.01X_{1}X_{3}-3.94X_{2}X_{3}–3.37X_{1}^{2}–0.135X_{2}^{2}–1.21X_{3}^{2} \end{array}$$

Based on the coded factor's coefficient, fat levels' effect on the WHC of cream cheese samples was more than basil and xanthan amounts (Table 3). The lowest WHC value (62.4%) was observed in cream cheese without basil seed and xanthan gum containing 16% fat. However, cream cheese with 24% fat containing 0.5 or 0.25% basil seed gum and 0.25% xanthan gum had the greatest WHC (100%). Collectively, with an increment of fat (X_1_) and basil seed (X_2_), and xanthan gum (X_3_) levels, WHC was significantly increased (Figure 1b1–b3). Our results regarding the influence of fat decrease and fat replacer incorporation on cheese WHC and moisture increment agree with the literature (Carocho et al., 2016; Naji‐Tabasi & Razavi, 2016, 2017; Ribas et al., 2019). WHC is an important characteristic of cream cheese and indicates the stability of the coagulation conditions of the cream cheese gel network.

### 3.2 Texture properties assessment

**FIGURE 2:**
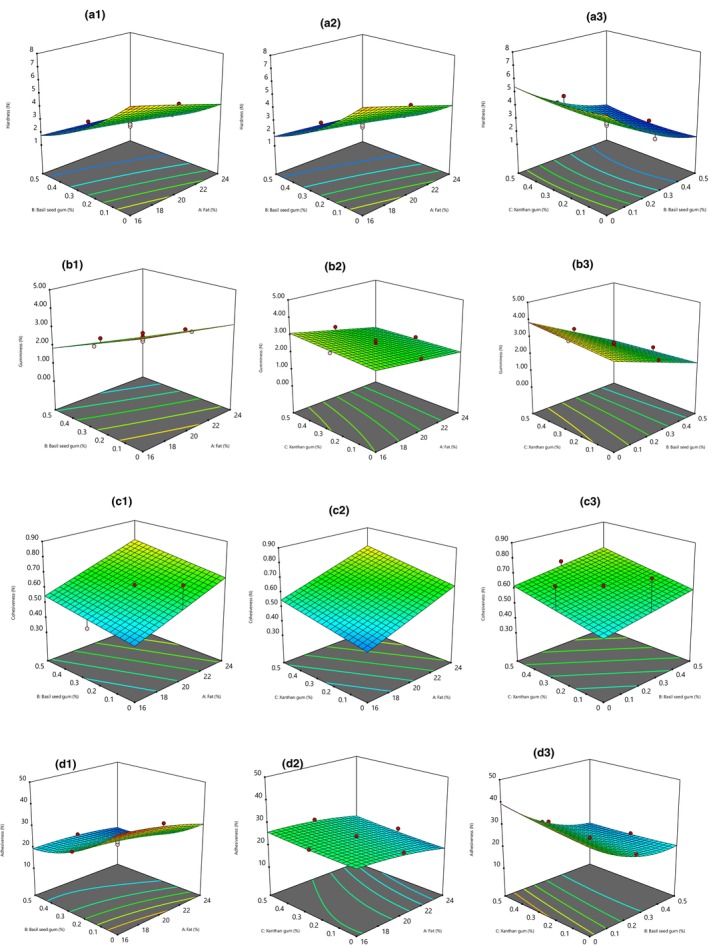
Response surface plot of the effects of basil seed and xanthan gum and fat content on the cream cheese texture properties including hardness (a1, a2, and a3), gumminess (b1, b2, and b3), chewiness (c1, c2, and c3), cohesiveness (c1, c2, and c3), and adhesiveness (d1, d2, and d3).

Hardness is the required force value to penetrate texture analyzer teeth into cheese. The hardness of cream cheese samples containing a higher fat level was significantly lower (*p* < .05). The fat reduction increased hardness while incorporating basil and xanthan into the cheese, resulting in reduced harness (Figure 2a1–a3). The data obtained by the design expert showed hardness of cheese samples followed a linear model and had a nonsignificant lack of fitness. The equation of hardness is included (Table 2).

(4) $$\begin{array}{c} \text{Hardness}=2.83–0.70{\text{2X}}_{1}–1.92{\text{6X}}_{2}-0.00{\text{9X}}_{3}+0.3675X_{1}X_{2}\\ +0.2325X_{1}X_{3}+0.10{\text{5X}}_{2}X_{3}+0.0186X_{1}^{2}+0.5514X_{2}^{2}+0.2464X_{3}^{2} \end{array}$$

With the fat reduction, the texture was tighter, although the addition of basil seed and xanthan gum caused moisture to entrap within the cheese was increased and created a softer texture (Sharafi et al., 2019). In the previous studies, the authors reported that various fat replacers such as xanthan, novel, galactomannan, basil, and WPC80 decreased cheese harness, similar to our finding (Aminifar et al., 2014; Rashidi et al., 2015; Ribas et al., 2019; Sharafi et al., 2019). Contrary to our results, Hosseini‐Parvar et al. (2015) found that an increment in the level of basil seed gum resulted in more hardness in processed cheese (Hosseini‐Parvar et al., 2015). Also, adding tragacanth gum into Lighvan cheese, konjac, and xanthan gums into spreadable processed cheese increased hardness and firmness (Ghods Rohani & Rashidi, 2019; Milani et al., 2017).

Gumminess was significantly and linearly influenced by dependent variables (fat content and basil seed, and xanthan gum concentration) employed (*p* < .05). The highest amount of gumminess (4.33 N) was found in cheese containing the lowest fat content (16%) without any basil and xanthan gum, while the lowest gumminess (0.5 N) was observed in samples with 24% fat and 0.5% basil seed and 0.5% xanthan gum. Based on the previous research, the fat reduction and incorporation of gums, including tragacanth and Simpless‐D 100, decreased cheese gumminess, and this finding was similar to our results (Milani et al., 2017; Romeih et al., 2002; Saint‐Eve et al., 2009). The results of this study were similar to other studies. For example, Nateghi (2020) showed that using xanthan gum and a low level of sodium caseinate reduced the gumminess in reduced‐fat cheddar cheese and mozzarella cheese (Nateghi, 2020). As well, the utilization of inulin in synbiotic UF soft cheese, basil essential oil in UF soft cheese, Persian and almond gums in low‐fat UF cheese, xanthan, and soy protein isolate in processed cheese, basil seed, and xanthan in Iranian low‐fat white cheese caused a decrease gumminess level (Abedini & Nateghi, 2017; Ghods Rohani & Rashidi, 2019; Hayam et al., 2017; Rostamabadi et al., 2016, 2017). Our results about reducing gumminess due to basil seed and xanthan gum were opposite to other research (Abiri & Bolandi, 2016; Ghods Rohani & Rashidi, 2019; Khani, 2019; Mahrooghi et al., 2017; Mohammadzadeh Milani et al., 2017).

Cohesiveness indicates the necessary force content for breaking of inner bond links of the product. The cohesiveness level was in the range of 0.36 to 0.89 N. As seen in Figure 2c1–c3, cohesiveness was directly related to fat reduction and the increment of basil and xanthan gum concentration. Therefore, the linear equation could significantly forecast the cohesiveness as a function of fat content and basil and xanthan gum level. This model is well fitted (*p* < .01, *F*‐value = 16.96) and also indicated as following:

(5) $$\text{Cohesiveness}\;\left(N\right)=0.6074–0.1089X_{1}+0.0486X_{2}+0.0604X_{3}$$

Sharafi et al. (2019) found that the fat reduction in ultra‐filtrated low‐fat cheese decreased cohesiveness, similar to our findings. However, the mentioned authors declared that the increasing galactomannan and novel content caused decreased cohesiveness. Also, Nateghi et al. (2012) found that changes in the cohesiveness of different reduced‐fat cheddar cheeses containing xanthan gum and/or sodium caseinate were non‐significant compared with the full‐fat control sample (Nateghi et al., 2012). Using β‐glucan in low‐fat cheddar cheese had the same cohesiveness behavior (Konuklar et al., 2004).

Adhesiveness is the degree of adhesion of the samples to the teeth, or in other words, the force required to separate the materials stuck to the mouth (usually the palate) during the normal process of eating. Another definition of adhesion is the work required to overcome the adhesion forces between the surface of the food and the surface of other materials with which the food is in contact (Baghdadi et al., 2018; Zheng et al., 2016). In the case of cream cheese, this property is considered one of the textural defects. Low‐fat cream cheese without gum had more adhesiveness value than high‐fat samples. In the low‐fat cream cheese, protein and moisture were higher. Therefore, protein‐water interactions were increased, and it caused enhancement of the adhesiveness (Nateghi et al., 2012). The results of this study showed that the addition of basil seed gum and xanthan gum reduces adhesiveness. The lowest adhesiveness (14.23 N) was observed in samples containing 0.5% basil seed gum, xanthan gum, and 24% fat. The variance analysis findings indicate that the quadratic effect of fat, basil seed gum, and xanthan gum on the degree of adhesiveness of the low‐fat cream cheese was significant (*p* < .05). The trend of the influence of all three independent factors on adhesiveness is inverse (Figure 2d1–d3). Hence the increment of the fat and both gums could reduce the adhesiveness. The basil seed and xanthan gum increased the product consistency, kept the protein‐fat matrix particles together more firmly, and prevented them from sticking to the surrounding environment. Our results were similar to the previous studies. For example, Baghdadi et al. (2018) found that incorporating basil seed gum into brined cheese could decrease adhesiveness (Baghdadi et al., 2018). Also, Salvatore et al. (2014) found that fresh cheese samples containing inulin had lower adhesiveness (Salvatore et al., 2014). The opposite of our findings, Ghods Rohani and Rashidi (2019) reported that adding konjac and xanthan to spreadable processed cheese increased adhesiveness (Ghods Rohani & Rashidi, 2019). Zheng et al. (2016) pointed out that the adhesiveness of sliced cheese was correlated positively with fat content (Zheng et al., 2016).

### 3.3 Sensorial properties

**FIGURE 3:**
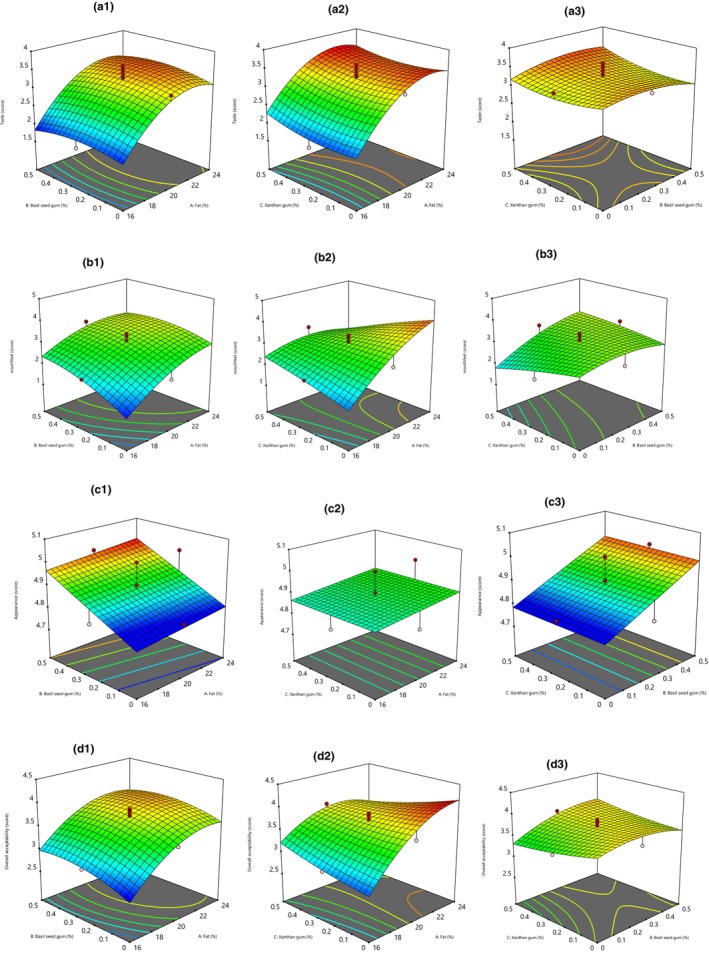
Response surface plot of the effects of basil seed and xanthan gum and fat content on the cream cheese sensory properties including taste (a1, a2, and a3), mouthfeel (b1, b2, and b3), appearance (c1, c2, and c3), and overall acceptance (d1, d2, and d3).

The sensory characteristics of cream cheese samples are shown in Figure 3. RSM model for the taste, mouthfeel, and overall acceptance of cream cheese was quadratic (Table 1) and significant. Results showed that fat reduction caused taste, mouthfeel, appearance, and overall acceptance score loss. Previous studies reported a loss of sensory properties for low‐fat cheese (Sharafi et al., 2019). Adding basil seed and xanthan gum could not significantly impact taste and appearance; therefore, reduced‐fat cream cheese had lower scores for the mentioned properties than samples with higher fat. Xanthan gum had not a significant effect on mouthfeel and overall acceptance score. At the same time, adding basil seed gum to fat‐reduced cream cheese could improve the score of mentioned attributes. The highest overall acceptance score (4.1) was related to cream cheese with 24% fat, and the sample containing 16% fat had the lowest overall acceptance score (26). The findings were similar to some previous studies. For example, Aydinol and Ozcan (2018) found that adding inulin and oat b‐glucan into reduced‐fat Labneh cheese caused better flavor than all the low‐fat cheeses (Aydinol & Ozcan, 2018).

### 3.4 Optimization

To produce low‐fat cream cheese, multi‐objective optimization was utilized to obtain a product with the least fat and the best physicochemical, texture, and sensory properties. After analyzing the data from 20 experiments with design expert software and achieving valid prediction models for each response, the software suggested a solution. Based on the optimal experimental conditions predicted by the face‐centered central composite design, 0.5% xanthan and 0.5% basil gum could manufacture a cream cheese with 19.04 fat. The fat level of this product was almost 20.67% lower than common cream cheese (24% fat), and it is considered safer cheese. Furthermore, the final price of the mentioned product was lower.

## 4 CONCLUSIONS

The present study aimed to produce low‐fat cream cheese with desired physicochemical, textural, and sensory properties by adding basil seed and xanthan gum. The RSM was applied to optimize fat level, basil seed, and xanthan gum content to achieve these goals. With the fat reduction of cream cheese, textural properties were lost, and the sensory score was decreased. However, adding basil seed and xanthan gum could improve these attributes. Collectively, RSM showed that the addition of basil seed (0.5%) and xanthan gum (0.5%) into cream cheese could manufacture a product with a lower fat level (19.04%) in comparison with common cream cheese (24%). In addition, its price was lower. The results of the present research could be utilized to produce low‐reduced‐fat cream cheese containing basil seed and xanthan gum with optimal quality at a commercial scale.

## AUTHOR CONTRIBUTIONS

**Jallal Portaghi:** Data curation (equal); investigation (equal); methodology (equal); writing – original draft (equal). **Ali Heshmati:** Conceptualization (equal); data curation (equal); formal analysis (equal); investigation (equal); methodology (equal); project administration (equal); resources (equal); software (equal); supervision (equal); validation (equal); visualization (equal); writing – original draft (equal); writing – review and editing (equal). **Mehdi Taheri:** Data curation (equal); investigation (equal); methodology (equal); project administration (equal). **Ebrahim eahmadi.eahmadi@basu.ac.ir Ahmadi:** Data curation (equal); formal analysis (equal); project administration (equal); supervision (equal). **Amin Mousavi Khaneghah:** Project administration (equal); supervision (equal); validation (equal); writing – review and editing (equal).

## CONFLICT OF INTEREST STATEMENT

The authors declare no competing interests.

## ETHICS STATEMENT

Before the study, the approval of the Research Ethics Committee of Hamadan University of Medical Sciences, Hamadan, Iran, for protocols and procedures utilized was obtained (Ethical code: IR.UMSHA.REC.1399.527).

## CONSENT

The authors declare their consent to publish this article.

## Data Availability

The data that support the findings of this study are available from the corresponding author upon reasonable request.
